# The Non-Haemostatic Response of Platelets to Stress: An Actor of the Inflammatory Environment on Regenerative Medicine?

**DOI:** 10.3389/fimmu.2021.741988

**Published:** 2021-09-13

**Authors:** Fabrice Cognasse, Hind Hamzeh-Cognasse, Patrick Mismetti, Thierry Thomas, David Eglin, Hubert Marotte

**Affiliations:** ^1^Etablissement Français du Sang Auvergne-Rhône-Alpes, Saint-Etienne, France; ^2^SAINBIOSE, INSERM, U1059, University of Lyon, Saint-Etienne, France; ^3^Vascular and Therapeutic Medicine Department, Saint-Etienne University Hospital Center, Saint-Etienne, France; ^4^Department of Rheumatology, University Hospital of Saint-Etienne, Saint-Etienne, France; ^5^Mines Saint-Étienne, Univ Lyon, Univ Jean Monnet, INSERM, U1059 Sainbiose, Saint-Étienne, France

**Keywords:** platelets, innate immunity, growth factors, inflammation, platelet-rich plasma

## Introduction

The development of platelet lysate (PL) or platelet-rich plasma (PRP) products to stimulate tissue repair and regeneration has been an important research field in various indications for more than 30 years (1–4), resulting in considerable interest in their potential for regenerative medicine. Moreover, many *in vitro*, *in vivo* and *ex vivo* studies have recently focused on the mechanisms of action by which these growth factors affect the biological activities of cells, thus supporting tissue healing. We feel that it is worth emphasising 1/the still limited understanding of their biological effect in the context of musculoskeletal injuries and other diseases, and 2/the lack of standardization of PL or PRP products. We also want to highlight the scientific cornerstones that need to be addressed to ensure ultimately safe and efficacious PL or PRP.

**Figure 1:**
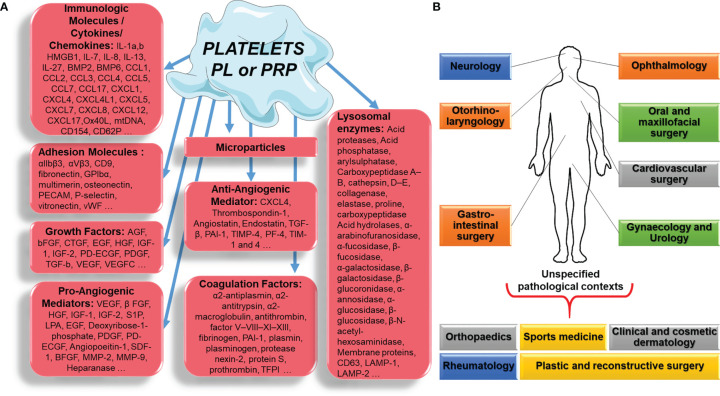
Platelet, platelet lysate (PL) or platelet-rich plasma (PRP) component **(A)**, applied in different pathological contexts **(B)**.

The vast majority of studies dealing with the efficacy of PL or PRP have focused only on the various growth factors - vascular endothelial growth factor (VEGF), insulin-like growth factor, fibroblast growth factor (FGF), platelet-derived growth factor (PDGF), brain-derived neurotrophic factor, transforming growth factor-beta (TGF-β), epidermal growth factor and others - that could play a role in the putative healing properties of PL or PRP through angiogenesis and cell stimulation. Surprisingly, while blood platelets are universally known for their major haemostatic role, as they restore vascular epithelial damage, their ability to produce and secrete a variety of cytokines, chemokines and related pro/anti-inflammatory molecules including growth factors, which contribute to this process and its integrated immune reaction **(****Figures 1A, B****)**, is rarely taken into account when deciphering the effects of PL or PRP administration.

We therefore believe that it is useful to draw a broader and ultimately more faithful picture of all the factors, platelets can release, with up to 1,100 proteins found in their secretome (5) that could trigger a mix of positive and negative effects on tissue healing, depending on the circumstances or local factors.

Platelets tightly regulate the process of angiogenesis (6, 7) by releasing both pro-angiogenic [VEGF, fibroblast growth factor-2, hepatocyte growth factor, PDGF, interleukin (IL)-8 and many others] and anti-angiogenic (thrombospondin-1, TGF-β, plasminogen activator inhibitor-1, IL-1-β and others) factors in α-granules (8). Moreover, proangiogenic factors such as VEGF are released in high concentrations in haemostatic clots, causing the proliferation and migration of proliferating endothelial cells (9). However, secreted anti-angiogenic factors such as TGF-β1 inhibit the proliferation and migration of endothelial cells. Platelet factor 4 (PF4), also known as chemokine (C- X-C motif) ligand 4 (CXCL4), is a small cytokine belonging to the CXC chemokine family. This chemokine is also released from activated platelet α-granules. PF4 has two important functions in the vasculature. It not only plays a proatherogenic role, but also has anti-angiogenic effects. It is suggested that the molecules released from the α-granules (*e.g.*, fibrinogen) and lysosomes of platelets participate in clot remodelling. Consequently, platelets and platelet-derived molecules participate in the natural process of wound healing and angiogenesis (8).

Platelets are also the main source of circulating microparticles (PMPs) (10). Circulating PMPs, present in PL or PRP, can release phosphatidylserine, which provides a highly procoagulant external surface. In addition, PMPs also release tissue factor, the main molecule in the coagulation cascade, and can deliver immunomodulatory factors such as CCL5, IL-1β, and CD40L, which modulate the activation of inflammatory cells such as neutrophils and are capable of exerting their pro-inflammatory activity outside of the blood compartment, thus linking thrombosis and inflammation (11). Platelets also release chemotactic molecules (IL-8, CCL-5, etc.) and other substances (especially immunomodulatory substances such as sCD40L), and trigger the proliferation of fibroblasts, endothelial cells and progenitor cells, thus regulating the healing process. It is important to emphasize again that many of the proteins and components of platelets are mechanically and chemically activated, and therefore their preparation can affect their efficacy enormously **(****Figure 1A****)**.

Recently, Jaron Nazaroff et al. published (12) a systematic review and Meta-Analysis (Cochrane Database, PubMed, and EMBASE databases) for level I/II clinical studies on PRP injections across all medical specialties [cardiothoracic surgery, cosmetic, dermatology, musculoskeletal (MSK), neurology, oral maxillofacial surgery, ophthalmology, and plastic surgery]. Generally, 61% of the studies found PRP to be favorable over control treatment, with no difference across medical specialties. The authors pointed to the inconsistent report of PRP throughout the medical research literature.

Because leukocyte is known to produce inflammation by driving the inflammatory phase of wound healing, research into leukocyte content in PRP formulations and the possible impact on osteoarthritis treatment has sparked some debate in the literature. However, José Fábio Lana et al. looked at the intriguing concept of leukocyte-rich platelet-rich plasma, or L-PRP, which may give benefits rather than drawbacks in terms of PRP’s regenerative potential (13).

## Concluding Remarks and Future Directions

PPL or PRP are currently recognized as products that promote tissue healing and regeneration. PRP is used in numerous areas, for acute diseases as much as for chronic diseases, for its haemostatic and healing properties in bone or soft tissue, by delivering supraphysiological concentrations of autologous platelets and their contents at the lesion site. We mainly use PL or PRP in tissue generation, due to the presence of high concentrations of growth factors.

Nevertheless, a wide variety cell adhesion molecule, cytokines, chemokines and integrins are also stored in platelets and secreted after activation. Additionally, by releasing various chemokines, PL or PRP recruit immune cells as neutrophils and monocytes/macrophages to establish a pro-inflammatory environment

New data suggest that PL and PRP mechanism of regenerative action depends on the pathological contexts: orthopaedics and sports medicine, rheumatology, oral and maxillofacial surgery, ophthalmology, otorhinolaryngology, clinical and cosmetic dermatology, plastic and reconstructive surgery, urology, neurology, gynaecology, gastrointestinal surgery and cardiovascular surgery **(****Figure 1B****)** (13).

Growth factors were previously assumed to be exclusively biological molecules that promote cell growth and proliferation, whereas cytokines were supposed to have solely an immunological or hematopoietic response. This view has changed over the past years and growth factors or cytokines can have diverse functions on different cell types, and multiple growth factors or cytokines can have similar or overlapping actions on different cell types.

The road to safe and efficacious PL or PRP: Standardized PRP/platelet lysate preparation with a potential adjunct delivery system to potentiate its effect (14). Identifying inflammatory parameters that may be linked to the donor, PRP (or equivalent), and patients would be beneficial to define how a patient can receive the most appropriate PRP or equivalent for their situation.

We can no longer exclude the inflammatory component and focus solely on the numerous or even a set of selected growth factors present in PL or PRP. Assessing the balance between growth factors with pro- or anti-angiogenic properties, as well as pro- or anti-inflammatory effects may provide a more personalized approach to identifying patients who may benefit from either PL or PRP, or an equivalent injection. Therefore, stratification and personalized medicine are ways to progress in the appropriate use of PL or PRP therapies.

## Author Contributions

FC and HH-C wrote the manuscript. All authors contributed to the article and approved the submitted version.

## Conflict of Interest

The authors declare that the research was conducted in the absence of any commercial or financial relationships that could be construed as a potential conflict of interest.

## Publisher’s Note

All claims expressed in this article are solely those of the authors and do not necessarily represent those of their affiliated organizations, or those of the publisher, the editors and the reviewers. Any product that may be evaluated in this article, or claim that may be made by its manufacturer, is not guaranteed or endorsed by the publisher.

## References

[1] Dos Santos RG Santos GS Alkass N Chiesa TL Azzini GO da Fonseca LF. The Regenerative Mechanisms of Platelet-Rich Plasma: A Review. Cytokine (2021) 144:155560. doi: 10.1016/j.cyto.2021.155560 34004552

[2] Giusti I D'Ascenzo S Macchiarelli G Dolo V. *In Vitro* Evidence Supporting Applications of Platelet Derivatives in Regenerative Medicine. Blood Transfus (2020) 18(2):117–29. doi: 10.2450/2019.0164-19 PMC7141937 31657710

[3] Etulain J. Platelets in Wound Healing and Regenerative Medicine. Platelets (2018) 29(6):556–68. doi: 10.1080/09537104.2018.1430357 29442539

[4] Andia I Abate M. Platelet-Rich Plasma: Combinational Treatment Modalities for Musculoskeletal Conditions. Front Med (2018) 12(2):139–52. doi: 10.1007/s11684-017-0551-6 29058255

[5] Senzel L Gnatenko DV Bahou WF. The Platelet Proteome. Curr Opin Hematol (2009) 16(5):329–33. doi: 10.1097/MOH.0b013e32832e9dc6 PMC2883290 19550320

[6] van der Meijden PEJ Heemskerk JWM. Platelet Biology and Functions: New Concepts and Clinical Perspectives. Nat Rev Cardiol (2019) 16(3):166–79. doi: 10.1038/s41569-018-0110-0 30429532

[7] Cognasse F Laradi S Berthelot P Bourlet T Marotte H Mismetti P. Platelet Inflammatory Response to Stress. Front Immunol (2019) 10:1478. doi: 10.3389/fimmu.2019.01478 31316518 PMC6611140

[8] Joshi S Whiteheart SW. The Nuts and Bolts of the Platelet Release Reaction. Platelets (2017) 28(2):129–37. doi: 10.1080/09537104.2016.1240768 PMC5446778 27848265

[9] Battinelli EM Markens BA Italiano JE Jr. Release of Angiogenesis Regulatory Proteins From Platelet Alpha Granules: Modulation of Physiologic and Pathologic Angiogenesis. Blood (2011) 118(5):1359–69. doi: 10.1182/blood-2011-02-334524. PMC3152500 21680800

[10] Puhm F Boilard E Machlus KR. Platelet Extracellular Vesicles: Beyond the Blood. Arterioscler Thromb Vasc Biol (2021) 41(1):87–96. doi: 10.1161/atvbaha.120.314644 33028092 PMC7769913

[11] van der Pol E Harrison P. From Platelet Dust to Gold Dust: Physiological Importance and Detection of Platelet Microvesicles. Platelets (2017) 28(3):211–3. doi: 10.1080/09537104.2017.1282781 28467294

[12] Nazaroff J Oyadomari S Brown N Wang D. Reporting in Clinical Studies on Platelet-Rich Plasma Therapy Among All Medical Specialties: A Systematic Review of Level I and II Studies. PloS One (2021) 16(4):e0250007. doi: 10.1371/journal.pone.0250007 33891618 PMC8064527

[13] Henschler R Gabriel C Schallmoser K Burnouf T Koh MBC. Human Platelet Lysate Current Standards and Future Developments. Transfusion (2019) 59(4):1407–13. doi: 10.1111/trf.15174 30741431

[14] Tang Q Lim T Shen LY Zheng G Wei XJ Zhang CQ. Well-Dispersed Platelet Lysate Entrapped Nanoparticles Incorporate With Injectable PDLLA-PEG-PDLLA Triblock for Preferable Cartilage Engineering Application. Biomaterials (2021) 268:120605. doi: 10.1016/j.biomaterials.2020.120605 33360073

